# Porous defective carbon ferrite for adsorption and photocatalysis toward nitrogen compounds in pre-treated biogas slurry

**DOI:** 10.1038/s41598-022-14772-z

**Published:** 2022-06-24

**Authors:** Jie Li

**Affiliations:** Institute of Agricultural Engineering and Technology, Fujian Academy of Agricultural Sciences, Fuzhou, 350003 China

**Keywords:** Pollution remediation, Structural properties

## Abstract

Carbon ferrite (C-Fe_3_O_4_) with hydrophilic functional groups and lattice defects was synthesized in anhydrous molten alkali system by fern leaves and ferric chloride as raw materials. Structural characterization results showed that carbon ferrite obtained oxygen-containing groups on the carbon surface. And structural pores and lattice defects resulted from spontaneous accumulation and “directive-connection” of ferrite (Fe_3_O_4_) nanoparticles. Carbon ferrite displayed an adsorption efficiency of 29.0% and excellent photocatalytic degradation of 80.8% toward nitrogen compounds (initial concentration of 430 mg/L) in pre-treated biogas slurry. The micromechanism for nitrogen compounds removal was discussed at the molecular/atomic level by exploring carbon ferrite “structure-activity”, which provides a design idea from microscopic perspective for the preparation of environmental materials with reactive sites.

## Introduction

At present, the livestock and poultry breeding industry still exposes some problems. High concentrations of nitrogen compounds (NC, 1500–3000 mg/L) containing organic amino acid and inorganic ammonia/nitrate nitrogen in biogas slurry wastewater have the probabilities for groundwater pollution and reductions in crop yield and quality^1^. Biogas slurry must be subjected to proper ripening treatment by microbial aerobic decomposition before being used in regional soil^2^. According to National Standards (GB 18,595-2001) for discharging sewage of livestock and poultry breeding, the safe concentration of ammonia nitrogen discharged into the environment is less than 80 mg/L, but microbial treatment techniques will be arduous to achieve. Photocatalytic technology for NC (ammonium/free ammonia) decomposition has many advantages since photocatalysts are not affected by the temperature, pH or toxicants in pre-treated biogas slurry^3–5^. NC are first adsorbed on photocatalyst surfaces though diffusion and electrostatic attraction and then decomposed by strong oxidative ·OH on active sites with final products that can only be N_2_, but the mechanism is not yet fully clear^5,6^.

Porous carbon sorbents can change the chroma and turbidity of biogas slurry; however, carbon has poor dispersibility in water due to fewer surface functional groups, and carbon surface hydrophilicity is primary for NC adsorption and photocatalysis^7^. Concentrated nitric acid/sulfuric acid is usually used to activate inert carbon to obtain more surface groups (–OH, –C = O). And concentrated alkali at 600–900 ℃ is adopted to introduce secondary pores via carbon surface corrosion^8^. Correspondingly, the activation processes are complex and expensive and require the equipment to be acid–alkali resistant and high-temperature resistant.

In this work, porous carbon with surface defects was prepared in a melted alkali system at 180 ℃ and atmospheric pressure with fern leaves as the carbon source. Some oxygen-containing groups retained after the synthetic reaction could improve carbon surface hydrophilicity and chemisorption. Bare carbon adsorbents are known to easily achieve adsorption equilibrium, and carbon powders dispersed in wastewater are difficult to post-treat and reutilize. Carbon has good conductivity but low quantum efficiency when used as a single component photocatalyst. Carbon nanocomposites are candidates for adsorption and photocatalysis. Ferrite (magnetic oxide) is a new photo-Fenton reagent with high catalytic activity and strong oxidation capacity. Ferrite absorbs visible light to generate electrons/holes to form strongly oxidized ·OH that can directly degrade NC, and ferrite is especially suitable for treating difficult biodegradable pollutants^9^. Carbon-iron oxide nanocomposites showed effective photodegradation of pollutants in wastewater^10^.

Many researchers have confirmed the positive roles of defective photocatalyst structures in photocatalytic processes^11–13^. Surface defects of ZnS synthetized by the salt wrapping method increased the number of reactive sites and the photocatalytic rate^14^. CeO_2_ obtained in organic solvents increased the adsorption capacity due to rich lattice defects^15^. Structural defects of MoS_2_ improved photocatalytic degradation activity by promoting charge separation, and active lattices of TiO_2_ could facilitate charge carrier scattering as an electronic driving force^16–18^, but their micromechanisms have rarely been explored. In this paper, by melted double bases as a reaction solvent without Ostwald ripening^19–21^, carbon ferrite (C-Fe_3_O_4_) was synthesized with fern leaves and iron trichloride as reaction materials, and ferrite nanoparticles formed cumulate holes and lattice defects by “directive connection”. Carbon ferrite showed synergistic effects by carbon adsorption and ferrite photocatalytic degradation toward NC in pre-treated biogas slurry, and their mechanisms have also been revealed at the microscopic level. In addition, magnetic ferrite can be used to separate carbon ferrite composites from wastewater for recycling.

## Results

### Morphology and microstructure

**Figure 1:**
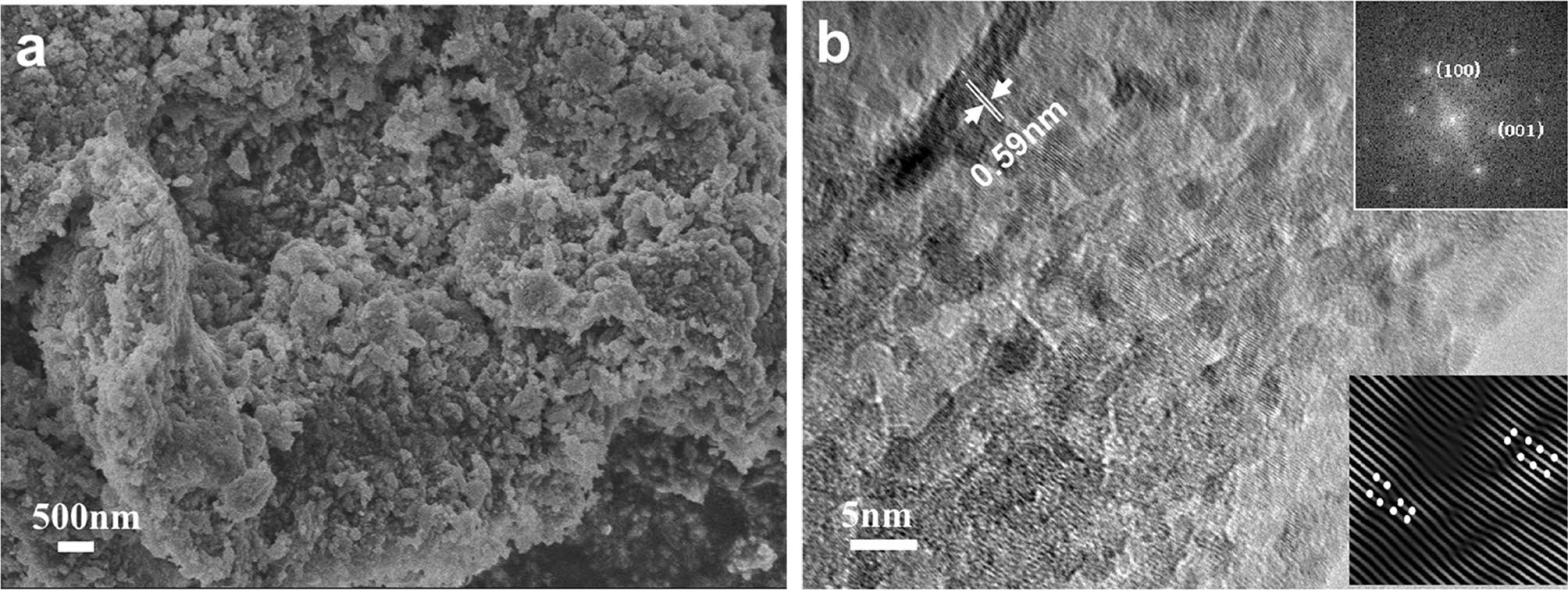
(**a**) SEM image. (**b**) TEM image of carbon ferrite and its corresponding FFT pattern (the inset in the upper right corner of Fig. 1b) and IFFT image (the inset in the lower right corner of Fig. 1b) of the Fe_3_O_4_ grains.

The SEM image of the carbon ferrite sample illustrated in Fig. 1a indicates tablet carbon with granular ferrite nanoparticles. The TEM image in Fig. 1b displays the nanocomposite microstructures in which carbon along the edge is amorphous and ferrite is the nanocrystallines of 5–10 nm scattered on carbon surfaces. The lattice fringes with a spacing of 0.59 nm correspond to the (100) facet of Fe_3_O_4_ according to the fast Fourier transform (FFT) pattern of the inset in the upper right corner of Fig. 1b. Several Fe_3_O_4_ grains have a similar orientation, with well-developed lattice fringes passing through them, as shown in the inset in the lower right corner of Fig. 1b. In anhydrous melted alkali solvent without Ostwald ripening, Fe_3_O_4_ nanoparticles tended to agglomerate to decrease the exposed surface to lower the surface energy^15,22^. The resulting nanocrystals easily bonded together if they were in a similar lattice orientation because the lattice mismatch energy at the interface would be reduced; thus, a spontaneously orientated attachment process occurred to Fe_3_O_4_ nanocrystals to form porous defective structures^15,23–25^.

### Components and pore structure

**Figure 2:**
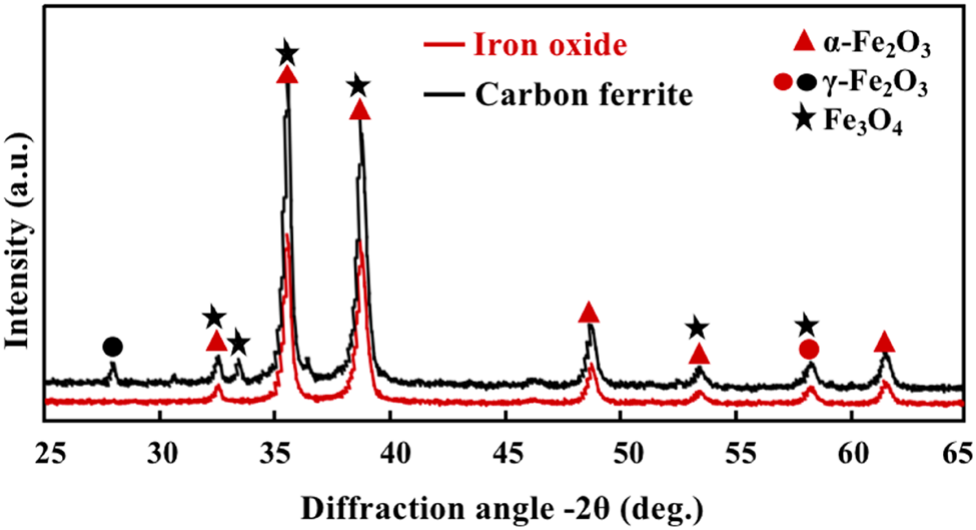
XRD patterns of iron oxide nanoparticles and carbon ferrite nanocomposites.

The XRD patterns of iron oxide and carbon ferrite are shown in Fig. 2. The XRD pattern of iron oxide displays peaks at 33.1°, 35.4°, 39.6°, 49.5°, 53.8°, and 62.4° corresponding to the (121), (110), (120), (220), (132), and (130) planes of α-Fe_2_O_3_ (JCPDS file No. 85-0978) and a weak reflection at 58.1° corresponding to the (511) plane of γ-Fe_2_O_3_ (JCPDS file No. 39-1346)^26,27^. Iron oxide synthesized in molten base system is the polycrystalline phase. Carbon ferrite was synthesized in the presence of ferric chloride reactant and carbon reductant by the melted alkalis method and calcination at 500 °C for 2 h in N_2_ atmosphere. The XRD pattern of carbon ferrite shows distinguishable peaks at 28.9°, 35.5°, 53.9°, 58.3°, and 62.3° corresponding to the (220), (311), (422), (511), and (440) planes of Fe_3_O_4_ (JCPDS file No. 85–1436) with a weak reflections at 34.4° corresponding to the (311) plane of γ-Fe_2_O_3_ (JCPDS file No. 25–1402) and three reflections at 33.1°, 39.7°, 49.5° corresponding to the (121), (120), and (220) planes of α-Fe_2_O_3_ (JCPDS file No. 85–0978)^27–30^. Fe_2_O_3_ was partly reduced to form Fe_3_O_4_ by carbon reductant in molten alkali liquid. Fe_3_O_4_ and γ-Fe_2_O_3_ are difficult to distinguish from the XRD patterns, and Fe_3_O_4_, α-Fe_2_O_3_ and γ-Fe_2_O_3_ could transformed in pairwise ways.

**Figure 3:**
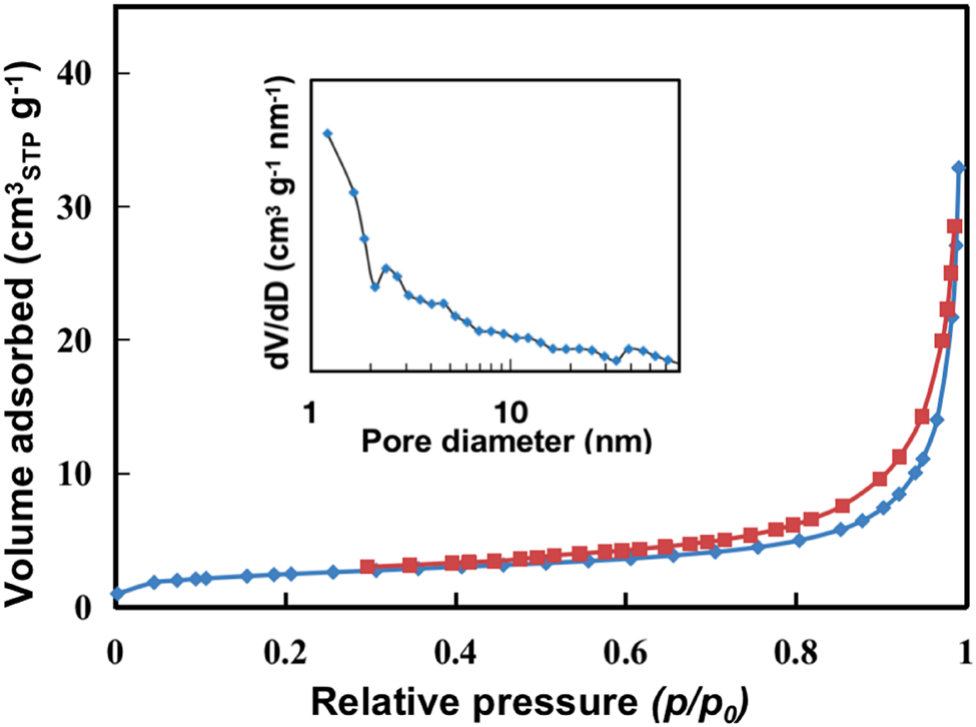
N_2_ adsorption–desorption isotherms of carbon ferrite and the inset showing the pore size distribution.

The nitrogen adsorption–desorption isotherm of carbon ferrite is a type IV isotherm with a typical hysteresis in Fig. 3. The hysteresis loop is a mixed type of H1 and H3, and the hysteresis loops of H1 and H3 formed by aggregations of uniform nanoparticles and flaky grains, respectively. SEM and TEM characterizations results indicated that the porous structures were produced by the coaggregation of ferrite nanoparticles and carbon nanosheets. The pore size distribution in the inset of Fig. 3 suggests that carbon ferrite had pore sizes from micropores to mesopores due to “close oriented attachment” of ferrite nanocrystals and carbon nano-blocks corrosion. Total pore volume of carbon ferrite was about 0.048 m^3^/g in Table 1, which is smaller than that of porous defective oxides prepared by the anhydrous system^14,15^, however, but the diameter of ammonia is only 0.5 nm. The micropores diameter and pore volume of carbon ferrite matched well the size of conical ammonia/ammonium, and NC in pre-treated biogas slurry could be adsorbed effectively and photo-degraded subsequently by carbon ferrite materials^4^.

**Table 1:** Summary of the physicochemical properties and the reutilization of the synthesized carbon ferrite.

Sample	S_BET_/(m^2^ g^-1^)	Mean pore diameter/nm	Total pore volume/(m^3^ g^-1^)	NC removal rate (the second cycle)	NC removal rate (the third cycle)
Carbon ferrite	8.79	21.9	0.048	65.9	46.8

### Surface and optical properties

**Figure 4:**
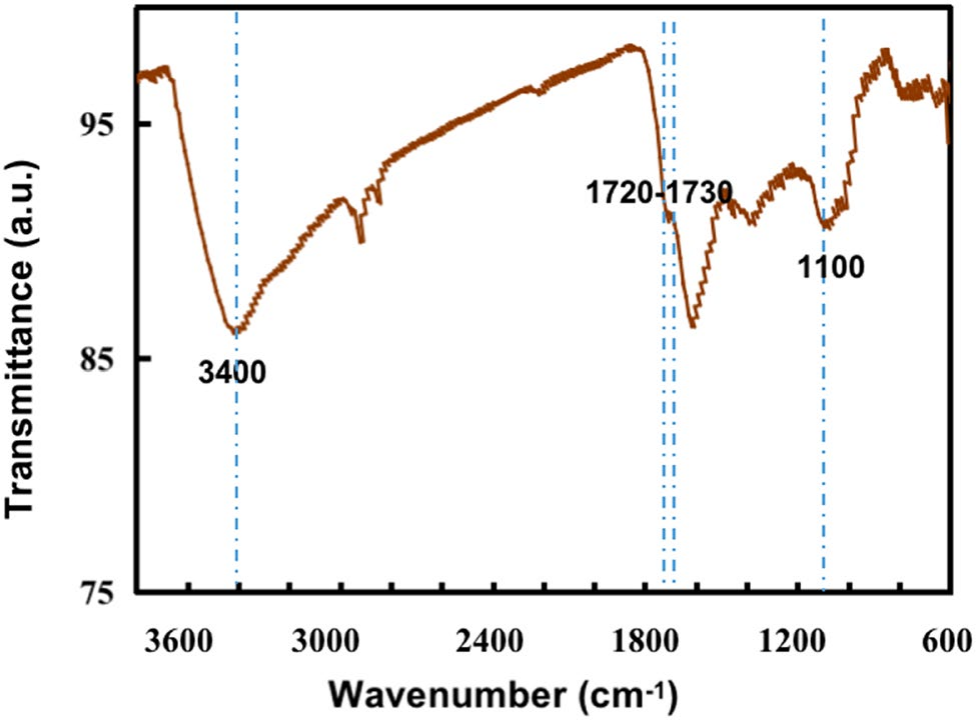
FT-IR spectra of the carbon ferrite sample.

**Figure 5:**
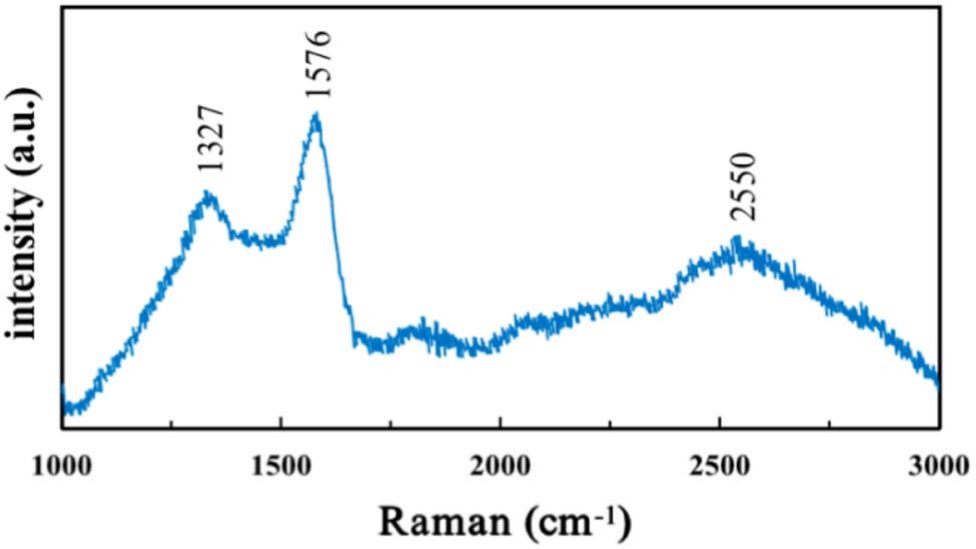
Raman spectra of the fern-leaf carbon sample.

**Figure 6:**
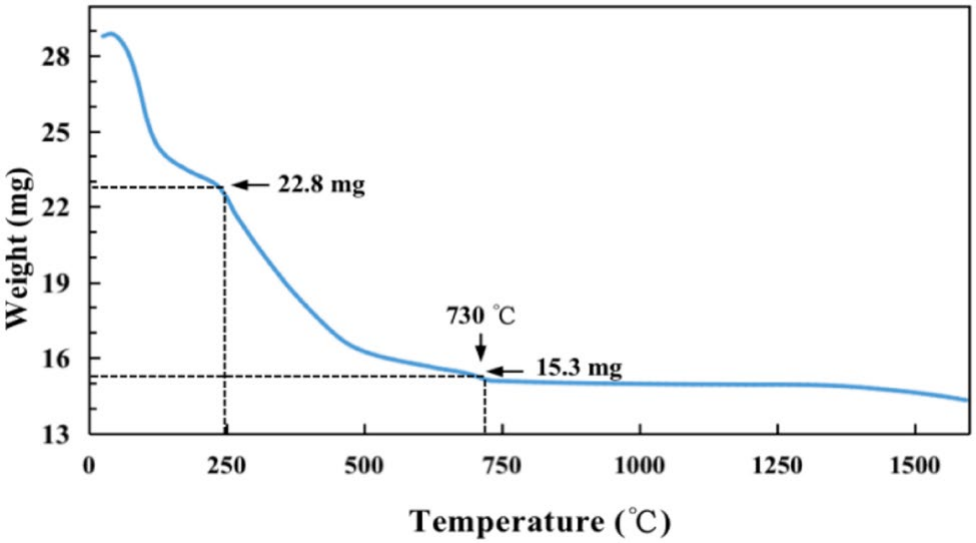
TG curve of the carbon ferrite sample.

The FT-IR spectrum of the carbon ferrite sample (Fig. 4) displays two characteristic peaks at 1100 and approximately 3400 cm^−1^ corresponding to the stretching mode of the OH groups. The bands at 1720 and 1730 cm^−1^ on the carbon surface demonstrate the vibrations of COOH groups, which assuredly means carbon ferrite has obtained oxygen-containing functional groups as fern leaves contain bioactive and optically active carboxyl-containing alkalis^31^. The Raman spectra showed the microstructure of fern-leaf carbon prepared by molten alkali method in Fig. 5. The 1327 and 1576 cm^-1^ features come from D and G bands, and the broad peak at 2550 cm^-1^ located in 2D-bank region. The D band corresponds to structural defects and G band is allocated to olefinic carbon structure^32,33^. Fern-leaf carbon obtained defective surface by base etching and oxygen-containing functional groups from fern leaf raw materials. The thermal stability and the component contents of carbon ferrite sample without pre-calcination were investigated by TG analysis as showed in Fig. 6. Weight loss of 6.008 mg was observed before 250 ℃ duo to the detachment of adsorbed water and fern-leaf carbon precursor decomposition. Fern leaf carbon was completed oxidized by oxygen and hydroxyl iron oxides changed to iron oxides between 250 and 730 ℃, resulting in 7.5 mg weight loss. After 730 ℃, weight change of the sample was attributed to the iron oxide transition. The final content of iron oxide in carbon ferrite composites was 67.1% and iron oxide displayed good thermal stability before 1500 ℃.

**Figure 7:**
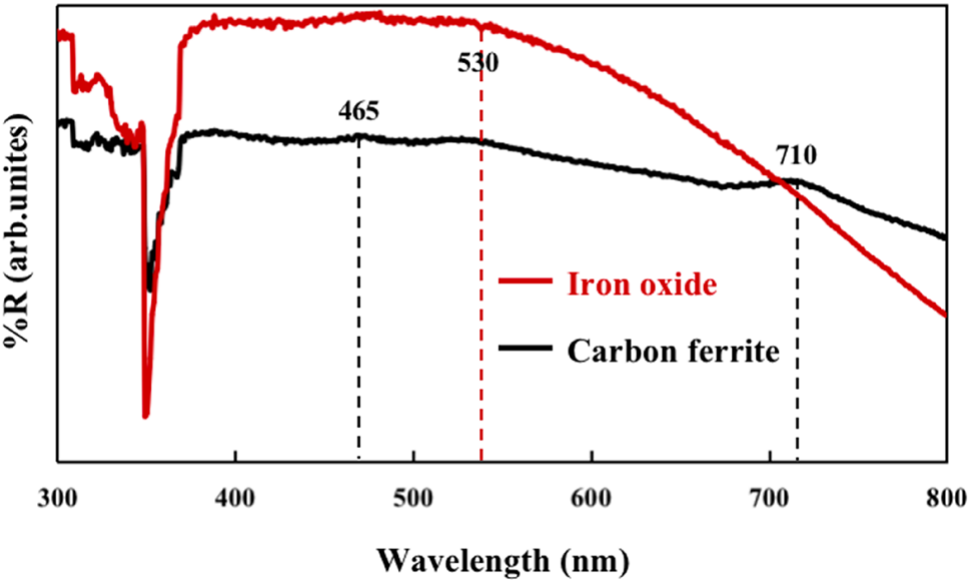
Diffuse reflectance spectra of iron oxide nanoparticles and carbon ferrite nanocomposites.

Figure 7 displays diffuse reflectance spectra of iron oxide nanoparticles and carbon ferrite nanocomposites. Iron oxide shows absorption of Fe_2_O_3_ between 465 and 530 nm. The carbon ferrite nanocomposites show an extended optical absorption in the visible region. Three absorption bands in the diffuse reflectance of the nanocomposites can be observed. The first two are observed at 465 and 530 nm, corresponding to the absorption of Fe_2_O_3_, and the third one at 710 nm is attributed to the absorption of Fe_3_O_4_ and the formation of Fe–O–C bonds between ferrite and carbon enhanced light absorption of the carbon ferrite nanocomposites^26,31^. The analysis of light absorption of the carbon ferrite sample is consistent with the XRD characterization result, with three types of iron oxide crystals corresponding to three optical absorption bands, showing the potential in optical application.

**Figure 8:**
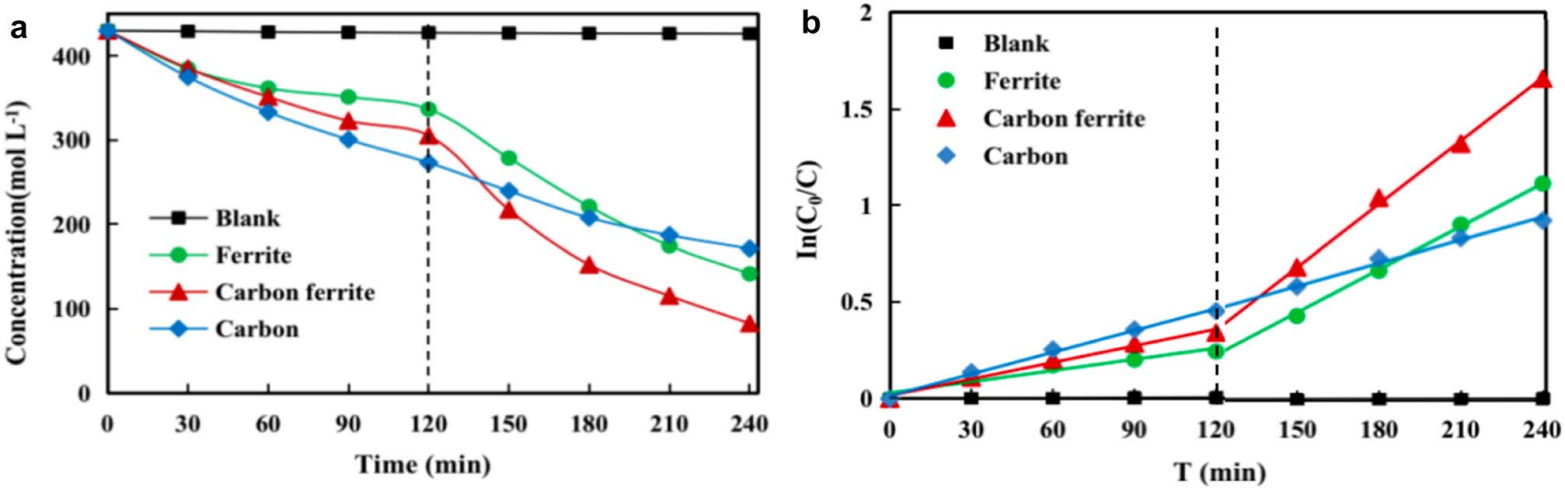
(**a**) Carbon ferrite adsorptivities (0–120 min) and photoactivities (121–240 min) for nitrogen compounds removal under visible light irradiation. (**b**) Plots of ln(C_0_/C) versus reaction time, showing the fitting results using the pseudo-first-order reaction.

### Adsorption and photocatalytic activity

The structural activity of the samples was tested by adsorption and photocatalysis toward NC in pre-treated biogas slurry. The reaction process can be expressed as a pseudo-first-order kinetic equation: ln(C_0_/C) = kt, where C_0_/C is the normalized NC concentration and k is the apparent reaction rate (min^-1^), and the sample activity has been defined as the corresponding reaction rate constant summarized in Table 2^26^ 36.0%, 21.7% and 29.0% NC were removed by fern-leaf carbon, iron oxide and carbon ferrite after adsorption in the dark, respectively (Fig. 8a, 0–120 min). Fern-leaf carbon is greater than iron oxide in adsorption capacity due to surface functional groups interacting with NC and abundant porosity by melted base etching, which is consistent with the k constants of the adsorption process (Fig. 8b, 0–120 min). The 60.2%, 67.1% and 80.8% NC were decomposed by fern-leaf carbon, iron oxide and carbon ferrite after visible light illumination (Fig. 8a, 120–240 min). The k constant (0.0108 min^-1^) for carbon ferrite is much higher than those for fern-leaf carbon (0.0039 min^-1^) and iron oxide (0.0073 min^-1^) (Fig. 8b, 120–240 min). Carbon ferrite sample appeared more prominent effect on NC removal than fern-leaf carbon and iron oxide in term of photocatalytic degradation efficiency. The circularizability of carbon ferrite sample was examined in Table 1. NC removal decreased by less than fifteen percent after the second usage and kept more than half of the original efficiency after the third cycle. Carbon ferrite material can be continuously reused for practicality for breeding sewage treatment.

**Table 2:** The physicochemical properties and adsorption/photodegradation rate constants of the synthesized carbon ferrite materials in melted bases after various reaction times and reaction temperatures.

Reaction time/min	Reaction temperature/°C	Crystallite size/nm	*D*_DFT_/nm	*k*_ads_/min^−1^	*k*_pho_/min^−1^
300	180	4.6	15.7	0.0015	0.0064
480	180	5.8	18.5	0.0019	0.0071
600	180	6.9	19.3	0.0023	0.0085
720	180	8.0	20.2	0.0038	0.0108
900	180	12.1	31.9	0.0029	0.0093

### Nitrogen compounds removal mechanism

**Figure 9:**
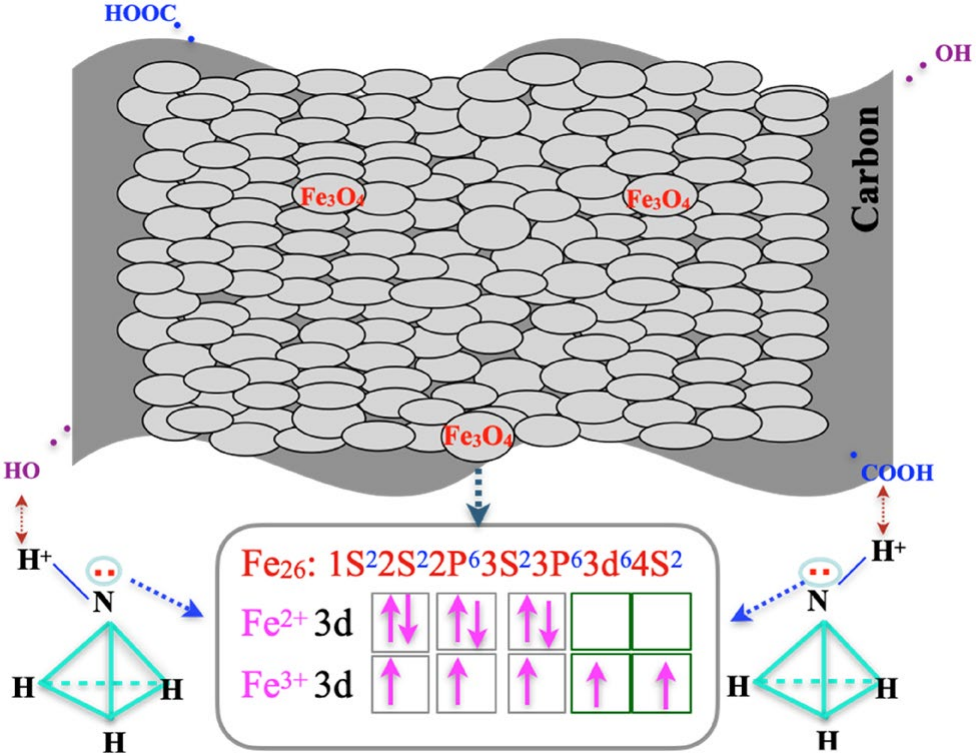
Schemata of the electronic structures of ammonium ion and iron oxide, and the electrostatic adsorption between NC and carbon ferrite.

**Figure 10:**
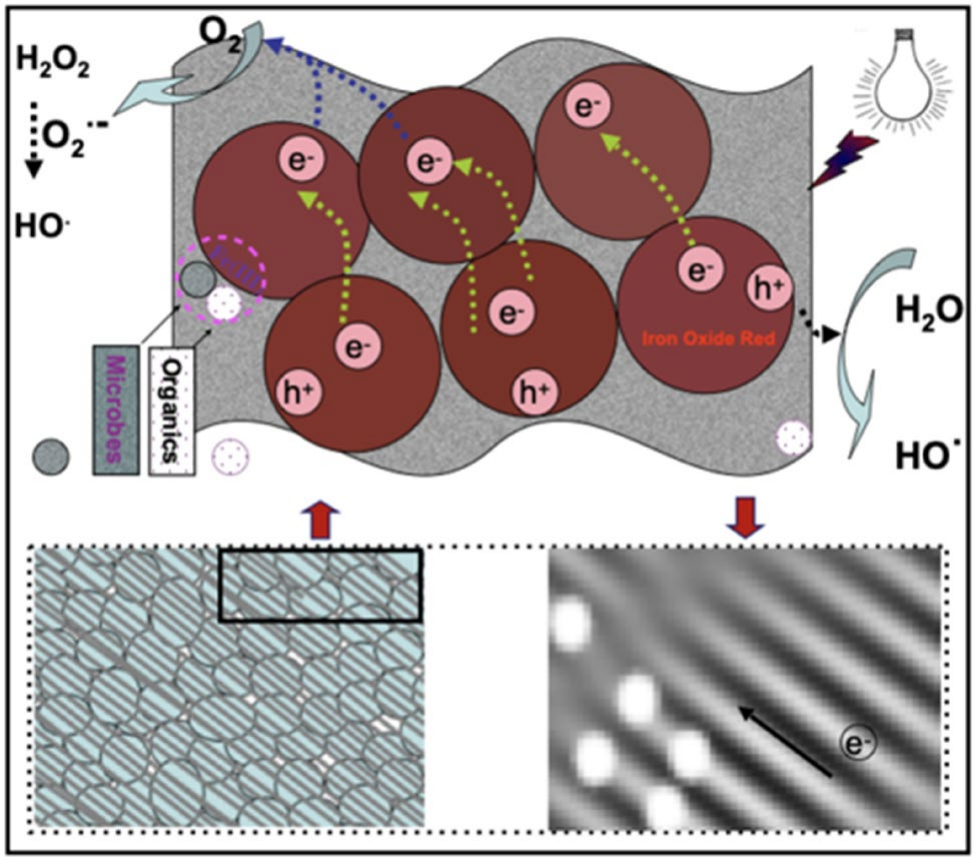
Schemata of the “Fe (III)-N-microbe” interaction relationship and nitrogen compounds photocatalytic mechanism.

Figure 9 exhibits the electronic structures of ammonium ion. Nitrogen atom of ammonium ion has a solitary pair of electrons and the iron atoms of iron oxide have 3d empty orbits, and the solitary pair of electrons could spread easily to the 3d free orbits of iron atoms to form a close connection. The H-bonds were produced by electrostatic interaction between the oxygen-bearing groups (-COOH, -OH) on the carbon surface and hydrogen ions of ammonium ions. NC pollutant molecules would been adsorbed by the pores physical function and active-sites chemical interaction. Fern-leaf carbon had high adsorption efficiency but low photocatalytic efficiency. Ferrite (Fe_3_O_4_) showed outstanding photocatalytic activity through guided-linking mismatched lattices (Fig. 1b) into the microscopic channels for transferring photoelectrons (Fig. 10)^18^. The microbial dissimilatory reduction process between microorganisms in pre-treated biogas slurry and ferrite formed “microbial-Fe(III)-N” interactions, as shown in Fig. 10. Therefore, carbon ferrite revealed a collaborative cycle effect on the removal of NC in pre-treated biogas slurry by carbon adsorption and ferrite degradation. This provides an idea for designing highly active photocatalysts by the main carbon ferrite structure-activity relationship and NC removal mechanism, which are both explored at the microscopic level.

### Concluding remarks

Carbon ferrite with accumulated pores and structural defects was synthesized by the melted alkali method. Functional groups on the surface of carbon improved water dispersibility and absorptivity, and lattice defects of ferrite increased reactive sites and microscopic channels for the photocarriers. Carbon ferrite displayed an adsorption efficiency of 29.0% and a degradation efficiency of 80.8% toward NC in pre-treated biogas slurry by carbon adsorbing, ferrite photodegradation and “microbial-Fe(III)-N” interactions. The research findings have positive significance to reduce nitrogen pollution in water and soil environments and promote the healthy development of livestock breeding.

## Materials and methods

Mixed sodium hydroxide and potassium hydroxide (AR, Jingke Chemical, Casma) were used as reaction solvents, and ferric chloride (AR, Xiongda Chemical, Casma) and fern leaf powder were treated by smashing and grinding ferns as raw materials. Carbon ferrite was obtained by the melted mixed bases method. One hundred grams of mixed hydroxides (mass ratio of NaOH/KOH = 1:1) were preheated to 180 ℃ for melting in a reaction kettle with a PTFE inner tank of 200 ml. And 28 g fern leaf powder with a mean diameter of 0.015 cm and 16.25 g ferric chloride were added to the molten hydroxides. The solid products were obtained after the 12 h reaction at 180 ℃ under normal pressure, washing using hydrochloric acid solution of pH = 1 and distilled water to neutral, and 2 h calcination at 500 ℃ in a N_2_ atmosphere. Fern-leaf carbon and iron oxide were synthesized separately via the melted bases method for comparison experiments.

The synthetic samples were characterized by X-ray powder diffraction (Philips PW3040/60), scanning electron microscopy (Hitachi S4800, Japan), transmission electron microscopy (JEOL 2100 F, Japan), Fourier transform infrared spectrometry (RAYLEIGH WQF-510A) at wavenumbers of 3800–600 cm^−1^, Raman spectrometer with laser wavelength of 532 nm (Labram HR Evolution, Horiba), and thermal weight analyzer (TGA/DSC 2 STAR System, Mettler Toledo) with a heating rate of 15 ℃/min in the oxygen atmosphere. N_2_ isotherms were recorded on a Quantachrome NOVA 2000e sorption analyzer, and the pore size distribution was based on the Barrett-Joyner-Halenda Model.

The initial concentration of nitrogen-containing compounds (NC) in biogas slurry from a local pig farm was 2800 mg/L. Biogas slurry was treated with bacterial carbon purification agent and the remaining NC (ammonium/free ammonia) concentration of pre-treated biogas slurry was 430 mg/L. The structural activity of synthetic samples was evaluated by the removal of NC in pre-treated biogas slurry with pH of 5–6. 800 mg of the sample and 800 ml of pre-treated biogas slurry (1 mg:1 ml) were placed in a 1000 ml tubular quartz reactor with a cooling water setting and irradiated by a 200 W lamp bulb as an illuminant (emission wavelength λ_max_ = 550 nm). Fifty milliliters of react liquid was sampled and measured at 30 min intervals by alkaline K_2_S_2_O_8_ Colorimetry (GB 11,894–89) on an SP-722 spectrometer for absorbance. Carbon ferrite photocatalyst was reactivated by washing in 2 M sodium hydroxide solution with stirring magnetically for 2 h. The recycling of carbon ferrite sample was investigated by reusing carbon ferrite powder separated magnetically from wastewater for adsorption and photocatalytic experiments as shown above.

## Data Availability

All data generated or analyzed during this study are included in this manuscript, and the datasets used and analyzed during the current study are available from the corresponding author on reasonable request.
